# The effect of spatial bottleneck on human eco-cultural range expansion

**DOI:** 10.1098/rsif.2025.1007

**Published:** 2026-10-21

**Authors:** Ryuichi Kumata, Seiji Kadowaki, Joe Yuichiro Wakano

**Affiliations:** ^1^ CEFE, CNRS, Univ Montpellier, EPHE, IRD, Montpellier, France; ^2^ Institute of Tropical Medicine, Nagasaki University, Nagasaki, Japan; ^3^ The University Museum, The University of Tokyo, Tokyo, Japan; ^4^ School of Interdisciplinary Mathematical Sciences, Meiji University, Tokyo, Japan

**Keywords:** cultural evolution, geographical boundary, reaction–diffusion model, travelling wave solution, blocking

## Abstract

The spatial spread of humans and culture is shaped by feedback between demography and cultural accumulation, as captured by the eco-cultural range expansion model. Yet, the effect of geographical boundaries on this process remains poorly understood. Here, we extend the model to a two-dimensional space where two large habitats are connected by a narrow corridor (spatial bottleneck) with reflecting boundaries. We find that a wave of ecological invasion always passes the bottleneck, whereas the subsequent transition to a high-density/high-culture equilibrium can be blocked by sufficiently narrow corridors. The maximum bottleneck width that prevents propagation depends on the domain shape, and it converges to the threshold value when the habitat to be invaded gets larger. Our results align with mathematical results on a bistable reaction– diffusion equation, particularly mean curvature flow, based on which we provide an approximate formula for the threshold bottleneck width. Applied to the Middle–Upper Palaeolithic transition, the findings suggest that while the spread of modern humans was robust to spatial bottlenecks, the expansion of advanced Upper Palaeolithic culture could be delayed or halted by narrow corridors. Archaeological records point to potential cases where such geographic bottlenecks constrained cultural dispersal.

## 1. Introduction

Recent archaeological and genetic studies suggest that the dispersal of *Homo sapiens* was shaped not only by biological adaptation but also by cultural processes [1–3]. A key context is the Middle to Upper Palaeolithic transition, when the spread of Upper Palaeolithic stone tool technologies coincided with the extinction of Neanderthals, who had coexisted with earlier *Homo sapiens* [4,5]. This archaeological overlap indicates that culture may have played an essential role in both the expansion of modern humans and the extinction of archaic populations.

Cultural evolution theory predicts the mutual interaction between population size and cultural level [6]. According to the population size hypothesis, larger populations are more likely to sustain and accumulate culture through social learning, and in turn, accumulated culture provides advanced technologies that support larger population sizes [6–8]. This feedback between population size and cultural level can create bistability between small populations with a low level of culture and large populations with a high level of culture [9]. Building on this feedback, the eco-cultural range expansion model was proposed to explain how demographic processes and cultural expansion interact during human dispersal [10,11]. The model predicts two types of waves in human expansion. The first, a monostable wave, advances by replacing an unstable equilibrium with a stable one– - corresponding to the replacement of archaic populations by a coexistence of archaics and moderns with a low cultural level. The second, a bistable wave, replaces one stable equilibrium with another—namely, the replacement of this coexistence by a large modern population with a high cultural level. The second wave is a coupled eco-cultural transition from a low-density/low-culture coexistence state to a high-density, high-culture modern-human state. This mechanism may account for archaeological observations: the initial colonization of *Homo sapiens* into Eurasia produced a transient coexistence with Neanderthals, followed by their extinction as *Homo sapiens* populations with high cultural levels expanded during the Middle-to-Upper Palaeolithic transition [10,11].

The geographical dispersal of human populations was constrained by uninhabitable regions such as seas, mountain ranges, rivers, and deserts [12]. As a result, humans often expanded through spatial bottlenecks formed by these geographic barriers. Examples of such bottlenecks include the Sinai Peninsula, which connects Africa and Eurasia [13]. Previous large-scale simulation studies, typically based on complex domains derived from global palaeoclimate models, have explored human dispersal under such settings [14,15]. However, these studies have commonly employed monostable reaction terms where the effect of spatial bottlenecks is relatively simple.

From a mathematical perspective, boundary conditions and domain shapes critically influence the qualitative behaviour of reaction–diffusion systems. This sensitivity is particularly pronounced in systems with bistable reaction terms, where domain shape can fundamentally alter the behaviours of the models. For example, stable non-uniform stationary solutions can emerge in non-convex domains [16,17]. Thus, since eco-cultural range expansion is essentially a bistable phenomenon, it is crucial to explicitly investigate how geographic bottlenecks shape its dynamics.

Here, we examine how the spatial domain affects eco-cultural range expansion. We investigate our eco-cultural range expansion in the specific spatial domains where two large habitats are connected by a narrow habitat with the reflecting boundary condition. We show that narrow bottlenecks can slow the second bistable wave without halting the geographic spread of humans. If the bottleneck is sufficiently narrow, the second wave associated with the transition to the high-density/high-culture modern-human state is blocked at its exit. The critical bottleneck width that blocks culture is negatively related to the speed of the second wave, consistent with mean curvature flow analysis. We discuss possible archaeological cases where such delays or blockings might have occurred.

## 2. Model

### 2.1. Basic model

To investigate the impact of spatial structure on the spread of human populations and their cultures in the geographical space, we extend the previous eco-cultural range expansion model [10,11] to that in a two-dimensional spatial domain Ω:

(2.1)∂N1∂t=rN1(1−N1+bN2M(Z1))+DΔN1,(2.2)∂N2∂t=rN2(1−N2+bN1M(Z2))+DΔN2,(2.3)∂Z1∂t=rZ1(1−N1+bN2M(Z1))−γZ1+δ(N1−Z1)+DΔZ1,(2.4)∂Z2∂t=rZ2(1−N2+bN1M(Z2))−γZ2+δ(N2−Z2)+DΔZ2

with Laplacian $\Delta :=\frac{\partial^{2}}{\partial x^{2}}+\frac{\partial^{2}}{\partial y^{2}}$ and with zero-flux boundary condition

(2.5) $$\begin{array}{r} \frac{\partial X}{\partial n}=0,\;X\in \{N_{1},N_{2},Z_{1},Z_{2}\},\;(x,y)\in \partial \Omega , \end{array}$$

where ∂/∂n is the derivative in the direction perpendicular to the boundary ∂Ω (the normal direction), representing the gradient along the normal vector. Our boundary condition assumes that humans do not migrate outside a certain habitable area across the boundary and that humans do not die because of migrating out of the habitable area.

The other variables and parameters follow the same assumptions as in Wakano *et al.* [10]. $N_{i}$ represents the density of species i, and $Z_{i}$ represents that of the skilled individuals of species i. r is the intrinsic population growth rate of humans, and $M(Z_{i})$ is the carrying capacity, which depends on the density of skilled individuals:

(2.6) $$\begin{array}{r} M(Z_{i})=\left\{ \begin{array}{clclc} & M_{H} & \; & Z_{i}>Z^{*} & \\ & M_{L} & \; & Z_{i}\leq Z^{*}. & \end{array}\right. \end{array}$$

Coefficient b is the interspecific competition coefficient. D is the diffusion coefficient of the hominoids. δ is the rate of skill acquisition and γ is the forgetting rate of the skills. Indices 1 and 2 refer to the resident archaics and the intrusive moderns. The carrying capacity depends on the density of skilled individuals (population size hypothesis) [6]. Note that each parameter ($r,b,M_{L},M_{H},Z^{*},\delta ,\gamma ,D$) is the same in both species, as we assume that the two species have the same cultural and ecological characteristics (i.e. the same reproductive rate, mobility, learning parameters). However, these parameters could be different between the two species. In the supplementary material, we discuss the asymmetric model in which the cultural and ecological parameters are different for each species.

### 2.2. Spatial domain

The spatial domain Ω consists of $\Omega_{1}$, $\Omega_{2}$ and $\Omega_{\text{SB}}$:

(2.7)Ω=Ω1∪ΩSB∪Ω2,(2.8)Ω1={(x,y)|0≤x≤L1,0≤y≤W1},(2.9)ΩSB={(x,y)|L1≤x≤L1+LSB,0≤y≤WSB},(2.10)Ω2={(x,y)|L1+LSB≤x≤L,0≤y≤W2}

(see figure 1). $\Omega_{1}$ and $\Omega_{2}$ are rectangular regions with lengths $L_{1}$ and $L_{2}$ in the x-direction and widths $W_{1}$ and $W_{2}$ in the y-direction, respectively. They are linked by a narrow corridor $\Omega_{\text{SB}}$. $\Omega_{\text{SB}}$ is an elongated rectangular region, representing the spatial bottleneck, with width $W_{\text{SB}}$ and length $L_{\text{SB}}$. We define $P_{1}$ and $P_{2}$ as the points exactly in the middle of the subdomains $\Omega_{1}$ and $\Omega_{2}$ (figure 1). In this study, we introduce modern humans to a part of $\Omega_{1}$ and investigate the spatial propagation of the eco-cultural transition through the areas already occupied by existing archaics.

**Figure 1.**
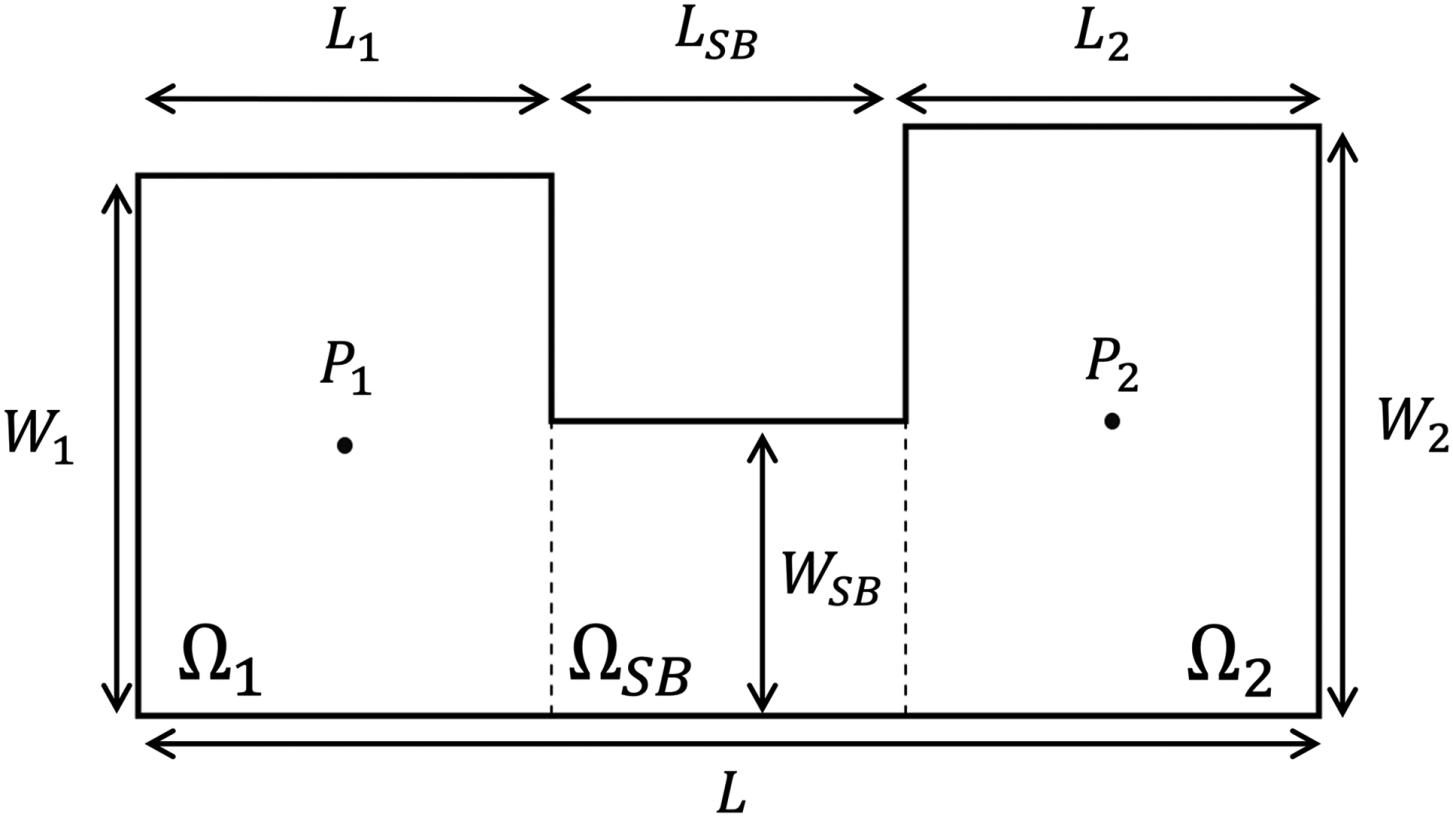
Spatial domain Ω. Two patches $\Omega_{1}$ and $\Omega_{2}$ are connected by a narrow corridor $\Omega_{SB}$. $L_{i}$ and $W_{i}$ are the length (horizontal) and width (vertical) of $\Omega_{i}$. $L=L_{1}+L_{SB}+L_{2}$ is the total length of the domain. $P_{1}=(x_{1},y_{1})=(L_{1}/2,W_{1}/2)$ and $P_{2}=(x_{2},y_{2})=(L-L_{2}/2,W_{2}/2)$ indicate the points located at the centre of $\Omega_{1}$ and $\Omega_{2}$.

### 2.3. Scaling time and space

To simplify the analysis, we consider the following rescaling:

(2.11) $$\begin{array}{r} \tilde{t}=rt,\;\tilde{x}=\sqrt{r/D}x,\;\tilde{y}=\sqrt{r/D}y. \end{array}$$

This scale transformation normalizes the diffusion coefficient to 1 and the intrinsic rate of human population growth to 1. With this scale transformation, the above systems are transformed to the following:

(2.12)∂N1∂t~=N1(1−N1+bN2M(Z1))+Δ~N1,(2.13)∂N2∂t~=N2(1−N2+bN1M(Z2))+Δ~N2,(2.14)∂Z1∂t~=Z1(1−N1+bN2M(Z1))−γ~Z1+δ~(N1−Z1)+Δ~Z1,(2.15)∂Z2∂t~=Z2(1−N2+bN1M(Z2))−γ~Z2+δ~(N2−Z2)+Δ~Z2,

where a tilde means the scale transformation and the spatial domain is also scaled to $\tilde{\Omega }$. In order to avoid complications in the notation, the tilde symbol is omitted hereafter.

### 2.4. Scaling density

Consider the transformation of the variables

(2.16) $$\begin{array}{r} {\hat{N}}_{i}=aN_{i},\;{\hat{Z}}_{i}=aZ_{i},\;{\hat{Z}}^{*}=aZ^{*},\;{\hat{M}}_{L}=aM_{L},\;{\hat{M}}_{H}=aM_{H}, \end{array}$$

then the equations with and without a hat become identical. This means that the model is invariant with the change of the unit of counting population density. To be more specific, the solution of a model with carrying capacity changing from $M_{L}$ = 20 $\text{people}\;{\text{km}}^{-2}$ to $M_{H}$ = 50 $\text{people}\;{\text{km}}^{-2}$ at a critical skilled density $Z^{*}$ = 15 $\text{people}\;{\text{km}}^{-2}$ is simply the halved population densities of the solution of a model with carrying capacity changing from $M_{L}$= 40 $\text{people}\;{\text{km}}^{-2}$ to $M_{H}$ = 100 $\text{people}\;{\text{km}}^{-2}$ at critical skilled density $Z^{*}$ = 30 $\text{people}\;{\text{km}}^{-2}$. Mathematically, this invariance of the dynamics under the scale transformation of density means that only the ratio $M_{H}:Z^{*}:M_{L}$ is meaningful for the dynamical outcomes of the system.

### 2.5. Reduced system

If the acquisition and loss of cultural skills are faster than the time scale of human reproduction and death, the skilled densities quickly converge to a local cultural equilibrium:

(2.17) $$\begin{array}{rl} Z_{1}(x,y,t) & =\theta N_{1}(x,y,t),\\ Z_{2}(x,y,t) & =\theta N_{2}(x,y,t), \end{array}$$

where

(2.18) $$\theta =\frac{\delta }{\delta +\gamma }$$

represents the proportion of skilled individuals at the cultural equilibrium. Moreover, if equation (2.17) is satisfied at $t=t_{1}$, then it holds for any $t\geq t_{1}$ (i.e. it is an invariant manifold of the original four-variable system) [10]. In this study, we assume this cultural equilibrium and consider the following two-variable system:

(2.19)∂N1∂t=N1(1−N1+bN2M(θN1))+ΔN1,(2.20)∂N2∂t=N2(1−N2+bN1M(θN2))+ΔN2.

The reduced model is characterized by five parameters: the interspecific competition coefficient b, the fraction of skilled individuals θ, the switching threshold $Z^{*}$ and the carrying capacities for high and low cultural levels, $M_{H}$ and $M_{L}$, respectively. Hereafter, we will study this system.

### 2.6. Equilibria without spatial structure

In the absence of spatial structure, there are in total eight possible equilibrium points with four internal equilibria:

(2.21) $$\begin{array}{r} \begin{array}{r} E_{LL}=\left(\frac{M_{L}}{1+b},\frac{M_{L}}{1+b}\right),\;E_{LH}=\left(\frac{M_{L}-bM_{H}}{1-b^{2}},\frac{M_{H}-bM_{L}}{1-b^{2}}\right),\\ E_{HL}=\left(\frac{M_{H}-bM_{L}}{1-b^{2}},\frac{M_{L}-bM_{H}}{1-b^{2}}\right),\;E_{HH}=\left(\frac{M_{H}}{1+b},\frac{M_{H}}{1+b}\right) \end{array} \end{array}$$

and four edge equilibria:

(2.22) $$\begin{array}{r} E_{L0}=(M_{L},0),\;E_{0L}=(0,M_{L}),\;E_{H0}=(M_{H},0),\;E_{0H}=(0,M_{H}), \end{array}$$

where the first and second quantities in the parentheses are the equilibrium densities of archaic and modern humans, respectively. The stability of these equilibria is classified by the parameter values of the system. If $M_{L}<Z^{*}/\theta <M_{H}/(1+b)$ and $0<b<M_{L}/M_{H}$, the four internal equilibria are stable and the four edge equilibria are unstable. On the other hand, if $M_{L}<Z^{*}/\theta <M_{H}/(1+b)$ and $b>M_{L}/M_{H}$, the two edge equilibria ($E_{0H},E_{H0}$) are stable instead of the two asymmetrical internal equilibria ($E_{HL},E_{LH}$). In the latter condition, one human species can be extinct (i.e. at $E_{0H}$ or at $E_{H0}$), or the two species can coexist (i.e. at $E_{LL}$, or at $E_{HH}$). In a previous study [10], it was demonstrated that the model under a one-dimensional spatial structure showed the dynamical replacement of archaic humans by modern humans through the spread of high-density populations of modern humans with the high cultural level (the replacement scenario). Here, we only consider the replacement scenario.

### 2.7. Numerical method

*Numerical simulation of partial differential equations*. For numerics, we adopt the alternating direction implicit method in the finite difference method. We first calculate changes by reaction terms by Δt/2, then calculate the diffusion in the x-direction by the implicit method. Next, we do the same for the y-direction. We set Δx=Δy=0.01 (for figure 2B) and Δx=Δy=0.05 (for the other figures). We set Δt=0.01 (for figures 2B, 3 and 4A,B) and Δt=0.1 (for figures 2A, 4C and 6).

**Figure 2.**
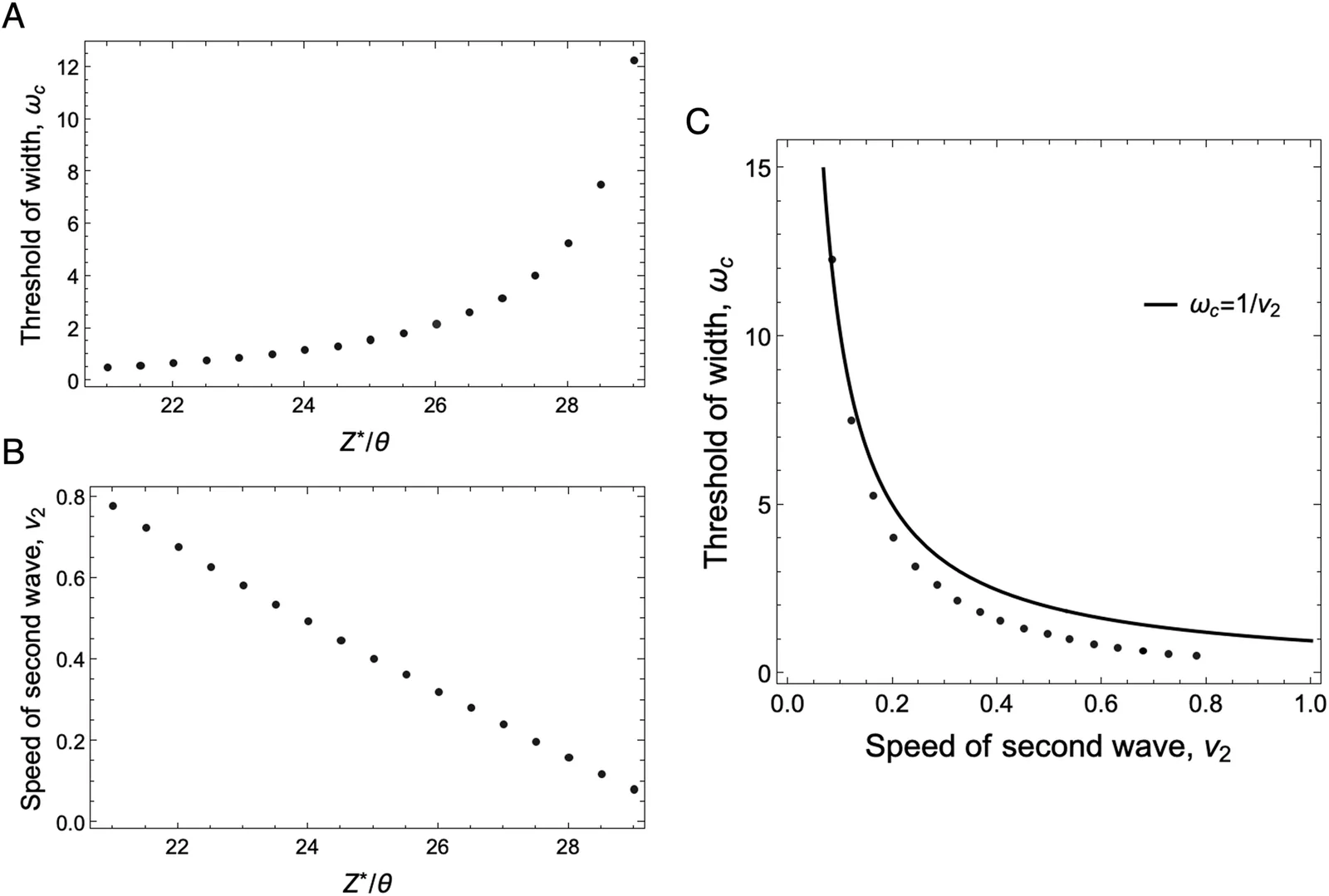
The effect of the speed of the second wave on $\omega_{c}$. (A) The dependency of $\omega_{c}$ on $Z^{*}/\theta$. The black dot indicates the data point of $\omega_{c}$. (B) The points show the speed of the second wave, $v_{2}$, which is numerically determined from the simulation in one-dimensional space. (C) The relationship between $\omega_{c}$ and the speed of the wave. The black line is given by the mean curvature flow model, $\omega_{c}=1/v_{2}$. The points are obtained by the combination of $\omega_{c}$ and $v_{2}$ for different $Z^{*}/\theta$. The basic domain shape: $W_{1}=24,W_{2}=24,L_{SB}=12,L_{1}=24,L_{2}=24$. Parameters are the same as in figure 3 (if not specified).

**Figure 3.**
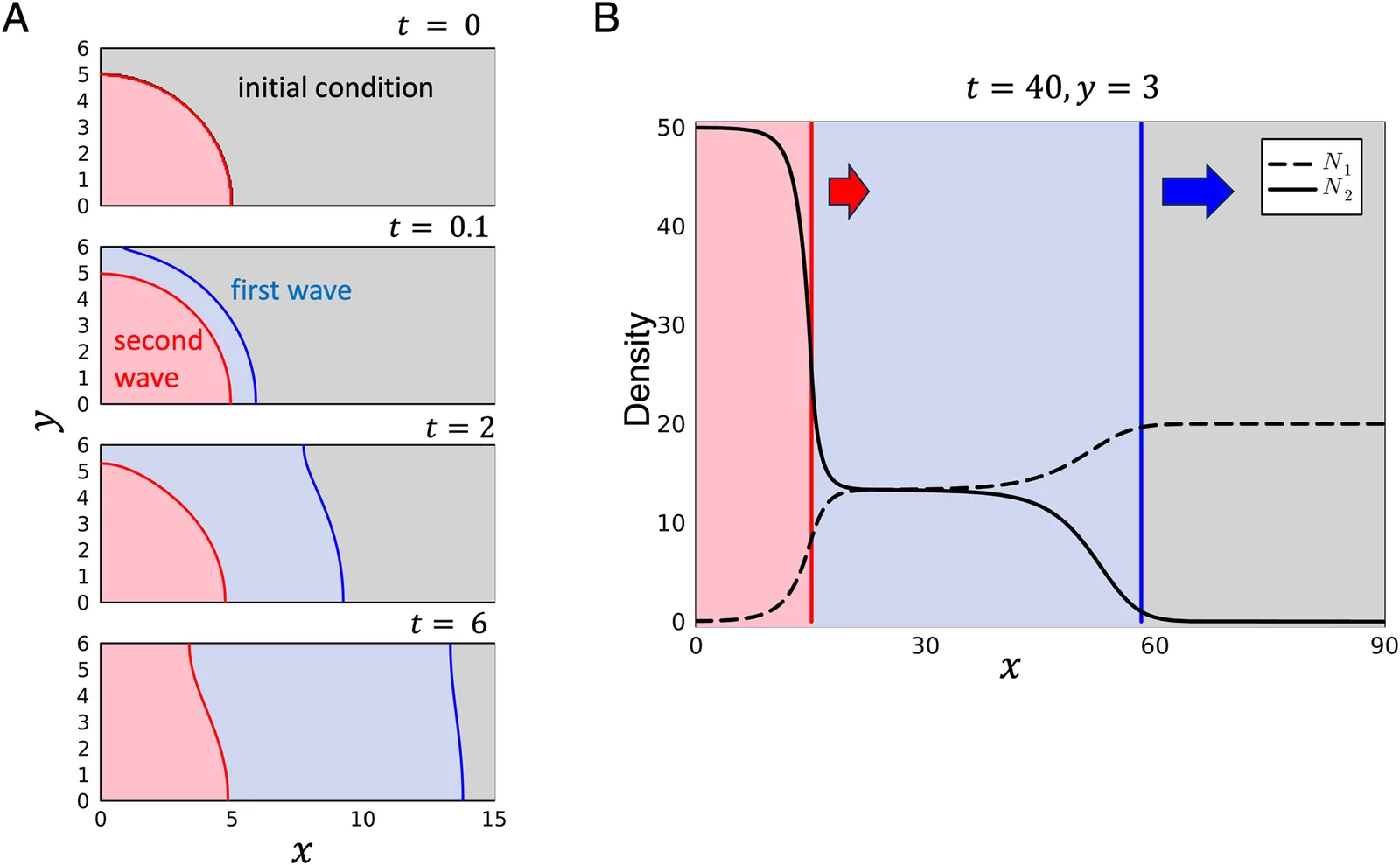
Spatial dynamics of eco-cultural range expansion. (A) Snapshots of the distributions. Each panel shows the regions where the modern human density is $N_{2}>Z^{*}/\theta$ (light red region), $1<N_{2}<Z^{*}/\theta$ (light blue region), and $N_{2}<1$ (light grey region) at the indicated time. (B) The wave profile at time t=40 and y=3. The black lines show the density of $N_{1}$ (dashed) and $N_{2}$ (solid). The vertical lines indicate the position of the fronts of the first wave (blue) and the second wave (red). Parameters: $Z^{*}=13,b=0.5,\theta =1/2,M_{H}=50,M_{L}=20$. The domain shape: $W_{1}=6,L_{1}=90,L_{\text{SB}}=L_{2}=0.$.

**Figure 4.**
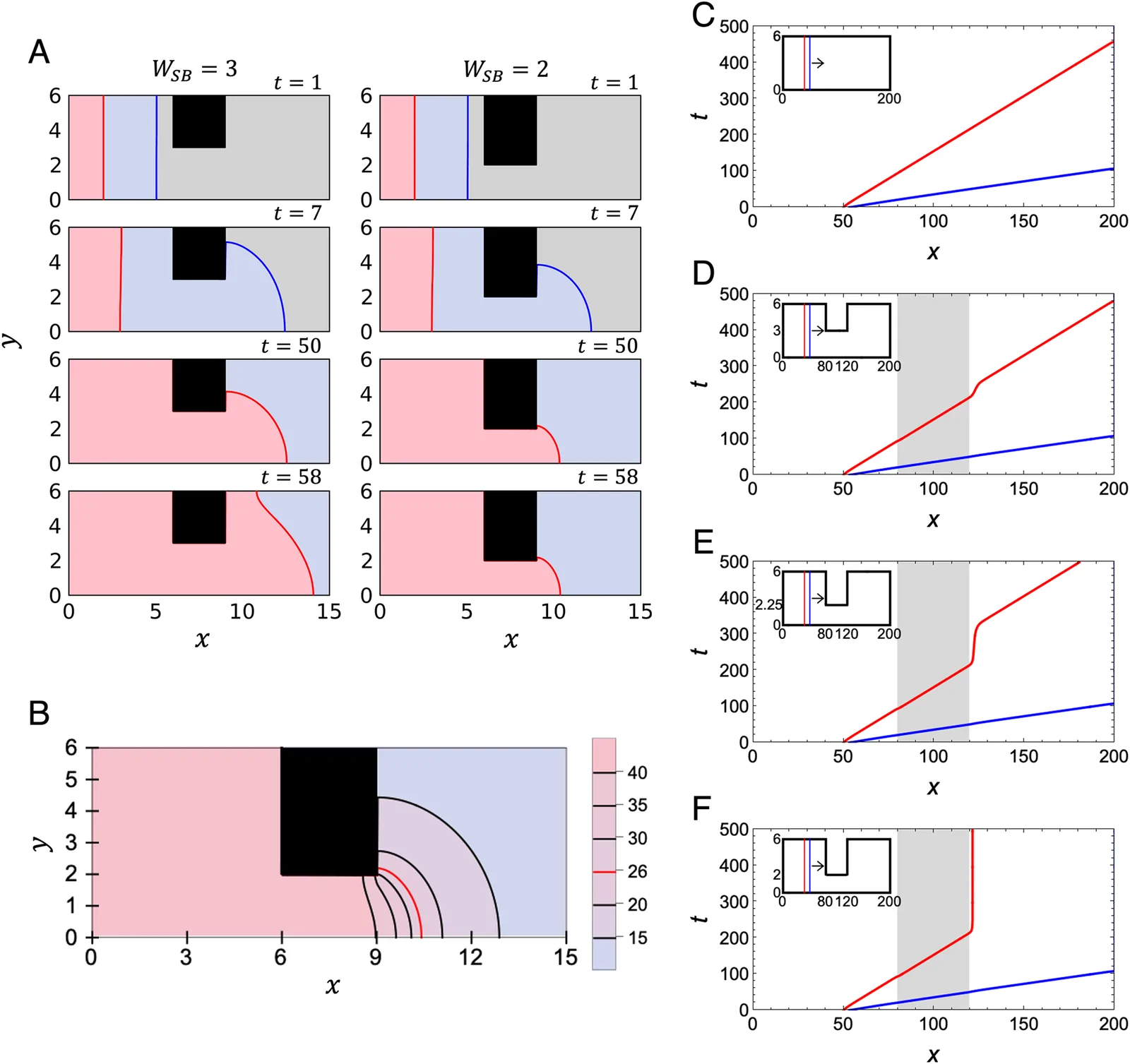
Narrow spatial bottleneck impedes eco-cultural range expansion. The blue curve indicates the position of the first wave ($N_{2}=1$), and the red curve indicates that of the second wave ($N_{2}=Z^{*}/\theta$). (A) Snapshots of the distributions at the indicated times with $W_{SB}=3$ (left) and $W_{SB}=2$ (right). (B) The density of modern humans $N_{2}$ at t=80. Black contours correspond to $N_{2}=40,35,30,20,15$ from left to right. (C–F) The position of wavefronts on the x-axis for the case without bottlenecks (C), with bottleneck width $W_{SB}=3$ (D), with bottleneck width $W_{SB}=2.25$ (E) and with bottleneck width $W_{SB}=2$ (F) in a longer domain. The wave position is calculated at y=1. The grey area represents the region of the bottlenecks. Parameters are the same as in figure 3. Domain shape in (A,B): $W_{1}=W_{2}=6,L_{1}=L_{2}=6,L_{\text{SB}}=3$. Domain shape in (C–F): $W_{1}=W_{2}=6,L_{1}=L_{2}=80\;\text{and}\;L_{\text{SB}}=40$.

**Figure 5.**
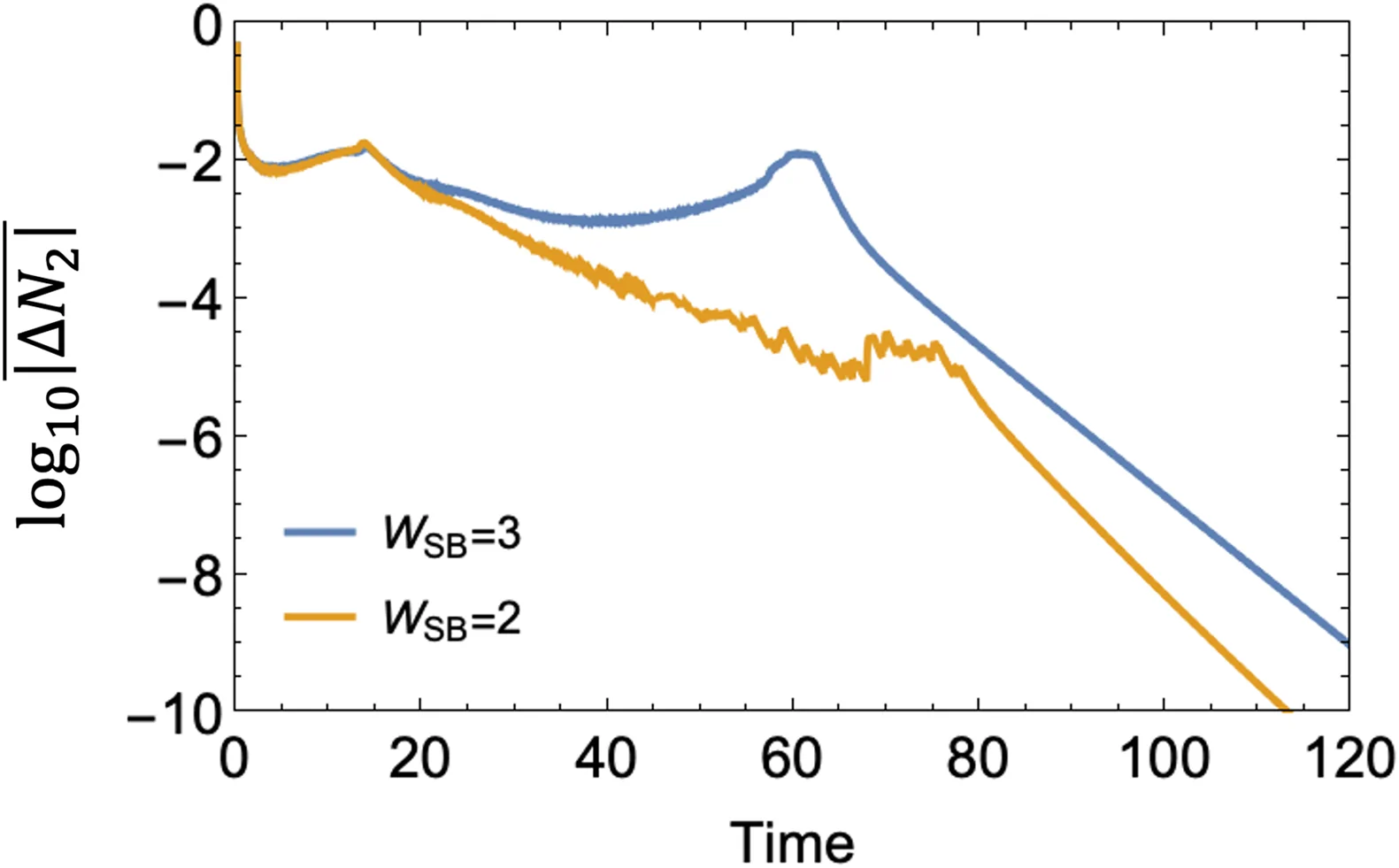
Dynamical change of $\overset{―}{\left|\Delta N_{2}\right|}$ during the eco-cultural range expansion dynamics in figure 4A.

**Figure 6.**
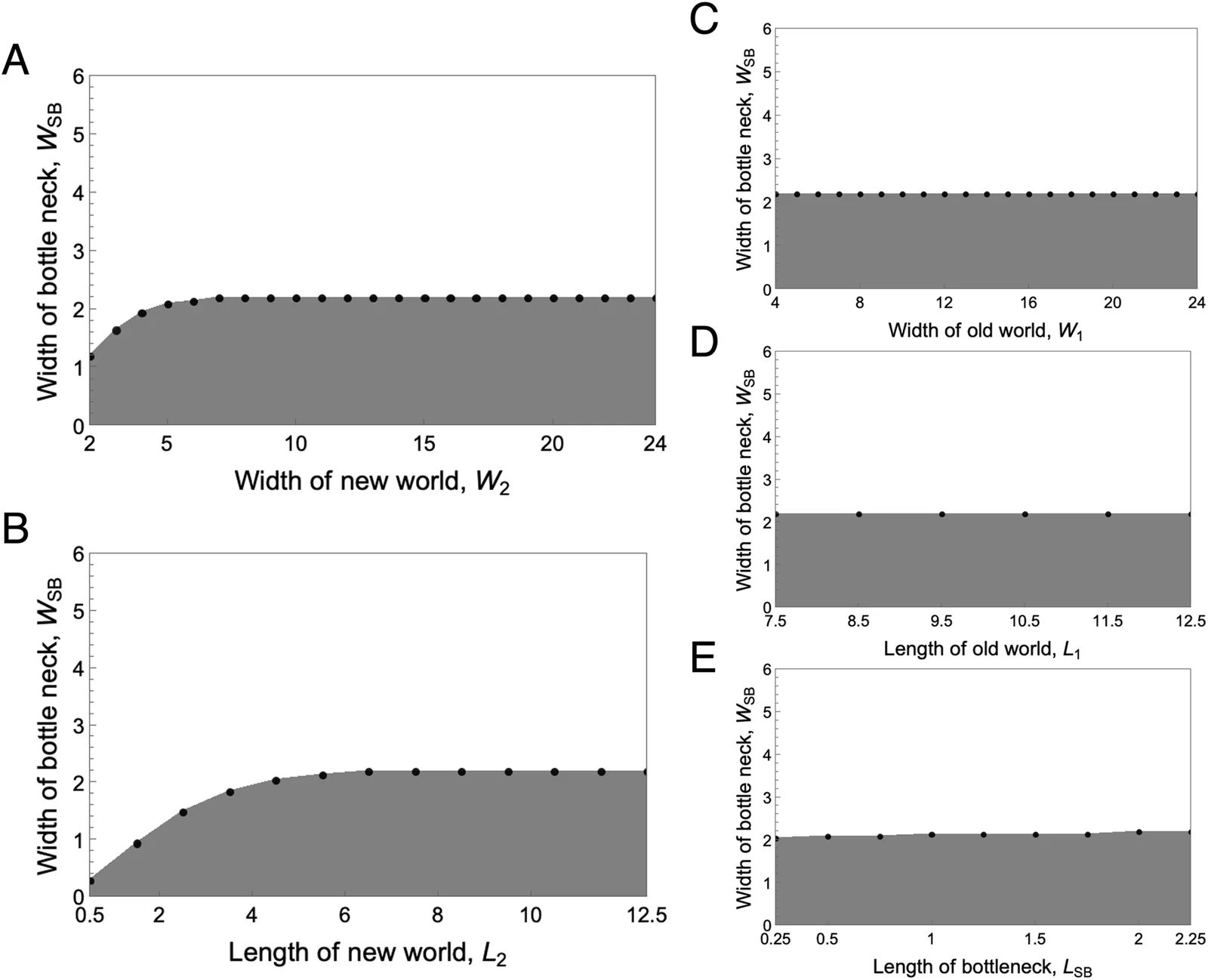
The dependence of domain shape on blocking. The black dot indicates the maximal width ω of the bottleneck that blocked the second wave. In the grey region, the second wave did not pass through the bottleneck. A black diagonal line in (A) indicates $W_{SB}=W_{2}$, above which $\Omega_{SB}$ is no longer a bottleneck. Parameters are the same as in figure 3. The baseline domain shape (if not specified): $W_{1}=24,W_{2}=24,L_{SB}=5,L_{1}=12.5\;\text{and}L_{2}=12.5$.

*Determination of the width of the bottleneck to stop the second wave*. At every integer time t≥1, we calculate $\overset{―}{\left|\Delta N_{2}\right|}$, the spatial average of the absolute values of the changes in density of $N_{2}$, where $\Delta N_{2}(x,y,t)=N_{2}(x,y,t)-N_{2}(x,y,t-1)$. We stop the simulation at time $\tilde{t}$ at which it drops below a certain threshold value for the first time ($\overset{―}{\left|\Delta N_{2}\right|}<10^{-6}$). We then determine whether the second wave is blocked or not by comparing the densities of $N_{2}$ at the representative points $P_{1}=(x_{1},y_{1})$ and $P_{2}=(x_{2},y_{2})$ at $t=\tilde{t}$ (see figure 1). If the densities of $N_{2}$ at $P_{2}$ are less than 95% of $N_{2}$ at $P_{1}$:

$$N_{2}(x_{2},y_{2},\tilde{t})<0.95\times N_{2}(x_{1},y_{1},\tilde{t}),$$

then we judge that the second wave is blocked by the bottleneck.

*Determination of the speed of the second wave*. We run the simulation of the system in a one-dimensional spatial domain as in [10]. We consider the space with length 400 with Δx=0.01 and Δt=0.1. With the following initial condition:

(2.23) $$\begin{array}{r} \begin{array}{rl} N_{1}(x,0) & =\left\{ \begin{array}{clclc} & 0 & \; & x\leq 40 & \\ & M_{L} & \; & x>40, & \end{array}\right.\\ N_{2}(x,0) & =\left\{ \begin{array}{clclc} & M_{H} & \; & x\leq 40 & \\ & 0 & \; & x>40, & \end{array}\right. \end{array} \end{array}$$

we measured the time and distance from the moment when the front ($N_{2}(x,t)=Z^{*}/\theta$) of the second wave passed x=200 in the simulation until it exceeded x=280.

## 3. Results

### 3.1. Rectangular spatial domain

Before examining the impact of spatial bottlenecks, we first show the behaviour of our model in the absence of bottlenecks ($W_{1}=6,L_{1}=90,L_{\text{SB}}=L_{2}=0$) (figure 3) with the following initial conditions:

(3.1) $$\begin{array}{r} \begin{array}{rl} N_{1}(x,y,0) & =\left\{ \begin{array}{clclc} & 0 & \; & \sqrt{x^{2}+y^{2}}\leq 5 & \\ & M_{L} & \; & \sqrt{x^{2}+y^{2}}>5, & \end{array}\right.\\ N_{2}(x,y,0) & =\left\{ \begin{array}{clclc} & M_{H} & \; & \sqrt{x^{2}+y^{2}}\leq 5 & \\ & 0 & \; & \sqrt{x^{2}+y^{2}}>5. & \end{array}\right. \end{array} \end{array}$$

In other words, the modern humans initially only inhabit the circular area with a radius of 5 centred on (0,0) (i.e. the lower left point of Ω) at high density $M_{H}$, while the rest of the area is occupied only by the archaic humans at low density $M_{L}$ (figure 3A top).

Figure 3A shows the snapshots of the two travelling wave fronts at different times. Similar to the one-dimensional case [10], the modern humans spread out by forming two types of travelling waves. The first wave replaces the unstable equilibrium $E_{L0}$ (in the grey area) where only the archaic humans exist with the stable equilibrium $E_{LL}$ (in the blue area) where the modern and archaic humans coexist at low density. The second wave replaces the stable coexistence equilibrium $E_{LL}$ (in the blue area) with a stable equilibrium where only modern humans exist at high density $E_{0H}$ (in the red area). We define the positions of the wavefront according to the density of modern humans. Here, the first wavefront (blue curve) is defined by the position of $N_{2}(x,y,t)=1$, and the second wavefront (red curve) is defined by the position of $N_{2}(x,y,t)=Z^{*}/\theta$ at which the carrying capacity switches. These two waves initially spread in two-dimensional space, forming spherical waves (t=0.1), and when they hit the boundary of $y=W_{1}$, the shape of the front is distorted and the direction of progression changes. These wave fronts converge to the planar travelling wave solutions that proceed along the *x*-axis (i.e. the position of the wavefront is no longer dependent on the *y*-coordinate).

Figure 3B shows the wave profile (population density of the modern and archaic humans) along the x-axis at $y=W_{1}/2$ at t=40, which looks identical to that of the one-dimensional case [10].

### 3.2. Effect of spatial bottleneck

We are interested in how the spatial bottlenecks affect eco-cultural range expansion. We consider the areas with longer length in the *x*-direction than width in the *y*-direction. In the following, we consider the initial conditions:

(3.2) $$\begin{array}{r} \begin{array}{rl} N_{1}(x,y,0) & =\left\{ \begin{array}{clclc} & 0 & \; & x\leq x_{0} & \\ & M_{L} & \; & x>x_{0}, & \end{array}\right.\\ N_{2}(x,y,0) & =\left\{ \begin{array}{clclc} & M_{H} & \; & x\leq x_{0} & \\ & 0 & \; & x>x_{0}, & \end{array}\right. \end{array} \end{array}$$

where the population of the moderns with a high culture ($x\leq x_{0}$) and the archaic population with a low culture ($x>x_{0}$) are separated at the boundary $x=x_{0}$. Hereafter, we use this initial condition unless we state the other conditions.

Two planar waves were rapidly formed and propagated in the x-axis direction in $\Omega_{1}$ (t=1 in figure 4A). Then, they entered the bottleneck $\Omega_{SB}$ one after another. Figure 4A shows the snapshots of cases with a wider ($W_{SB}=3$) and a narrower ($W_{SB}=2$) bottleneck. In both cases, the first wave (blue line) went through the bottleneck and propagated in $\Omega_{2}$. However, the second wave was blocked at the end of the narrower bottleneck ($W_{SB}=2$). Figure 4B shows the contour plot of the modern human density at t=80 with the narrow bottleneck. A relatively gradual spatial cline was observed at the exit of the bottleneck. To check if this heterogeneous spatial pattern is stationary, we tracked the changes in the distribution of $N_{2}(x,y,t)$ and confirmed that the rate of change was exponentially declining (figure 5, see t>80).

The dynamics of the positions of the two fronts at y=1 in four types of large domains ($W_{1}=6,W_{2}=6,L_{1}=80,L_{SB}=40,L_{2}=80$ and $W_{SB}=2,2.25,3,6$) are shown in figure 4C–F. The initial condition is given by equation (3.2) with $x_{0}=50$. Without a spatial bottleneck (figure 4C), the first and second waves quickly reached their own speeds and the first wave is faster than the second wave. In the case with a wide bottleneck ($W_{SB}=3$), the first wave (blue) was almost unaffected, while the dynamics of the second wave (red) was largely affected by the bottleneck (figure 4D). The second wave continued to travel at the same speed inside the bottleneck, but at the exit of the bottleneck it slowed down suddenly at t∼200. It took some time until it restarted to travel at t∼250. When the wavefront departed the bottleneck, the wave recovered the same speed as before and continued spreading in $\Omega_{2}$. The delay in the progress of the second wave at the exit of the bottleneck becomes longer with the narrower bottleneck ($W_{S}B=2.25$, figure 4E). For an even narrower bottleneck ($W_{SB}=2$), the second wave did not pass through the bottleneck and came to a complete stop near the end of the bottleneck (figure 4F). This is in sharp contrast to the first wave, which is barely affected by the bottleneck.

Numerically observed blocking implies a rapidly growing waiting time until the second wave restarts or the existence of a stable heterogeneous stationary solution in our system (see the Discussion for mathematical details). In the application to human dynamics, they share the same implications: the spatial bottleneck does not block the diffusion of modern humans (first wave) themselves, but it can block the spread of high density of modern humans and their advanced culture (second wave).

### 3.3. Effect of domain shape

Our numerics so far suggested the existence of a maximum width ω such that for any $W_{SB}\leq \omega$ the second wave is blocked. We studied the effect of domain shape on ω (figure 6). The baseline parameters were $W_{1}=24,W_{2}=24,L_{SB}=5,L_{1}=12.5\;\text{and}\;L_{2}=12.5$, and the initial values were equation (3.2) with $x_{0}=5$.

First, we investigated the effect of the domain shape of the new world, $\Omega_{2}$ (figure 6A). We found that ω is an increasing function of $W_{2}$ and $L_{2}$. Importantly, we found that ω converged to the critical width $\omega_{c}$ (around 2.2 under the parameter values used in these simulations) when $W_{2}$ and $L_{2}$ are sufficiently large. We also found that ω hardly depends on the shape of $\Omega_{1}$ (i.e. $W_{1}$ and $L_{1}$) and the length of the bottleneck, $L_{SB}$ (figure 6B). The stronger dependence of ω on the geometry of $\Omega_{2}$ can be understood as follows. For the domain sizes considered here, the source habitat and the corridor are long enough for the incoming front to converge to an approximately planar wave before reaching the corridor exit. Consequently, the detailed geometry of $\Omega_{1}$ and the bottleneck length have little effect on the blocking threshold. By contrast, the geometry of $\Omega_{2}$ directly constrains the curvature and relaxation of the front emerging from the corridor. As $\Omega_{2}$ becomes sufficiently large, these downstream boundary effects diminish and the critical width converges to its asymptotic value. In conclusion, we found the critical threshold of bottleneck width $\omega_{c}$ if $W_{2}$ and $L_{2}$ are sufficiently large.

### 3.4. The relationship between the speed of the second wave and the critical width $\omega_{c}$

In our system, $Z^{*}/\theta$ represents the critical population density that switches the carrying capacity of the population, and hence it plays a key role in shaping the speed and direction of the second wave in the one-dimensional space. We computed the critical bottleneck width $\omega_{c}$ across a range of $Z^{*}$ values ($10.5\leq Z^{*}\leq 14.5$ by 0.25) with θ=0.5. As a result, $Z^{*}/\theta$ varied from 21 to 29. We found that $\omega_{c}$ increased with $Z^{*}/\theta$, indicating that higher $Z^{*}/\theta$ values make the second wave more easily blocked (figure 2A). We also measured the speed of the second wave $v_{2}$ in the corresponding one-dimensional setting (figure 2B). As detailed in the previous study [10], the speed $v_{2}$ is a decreasing function of $Z^{*}/\theta$ (figure 2C).

#### 3.4.1. Mean curvature flow

Previous mathematical studies on travelling waves in reaction–diffusion models give some clues to understand these behaviours. In reaction–diffusion systems in two-dimensional space, the curvature of a travelling wavefront can affect its propagation speed. When the wavefront is only gently curved, so that its radius of curvature is much larger than the width of the transition layer, each point on the front can be approximated locally as a one-dimensional planar travelling wave. Under this assumption, the velocity in the normal direction of a curved wavefront follows the linear eikonal equation [18,19]. Based on the linear eikonal equation, when both diffusion coefficients are equal and rescaled to D=1, the motion of the wavefront is expressed as

(3.3) $$V(\vec{x},t)=V_{0}-\kappa (\vec{x},t),$$

where $V(\vec{x},t)$ is the normal speed of the wavefront at position $\vec{x}$, $\kappa (\vec{x},t)$ is the mean curvature of the wavefront, and $V_{0}$ is a constant determined by a reaction term. By definition, $V_{0}$ is the speed of the travelling wave in the corresponding one-dimensional system. This curvature-dependent velocity law is closely related to mean curvature flow (MCF), where an interface moves with normal velocity affected by its mean curvature, a type of curvature-driven motion that also arises as the sharp-interface limit of bistable reaction– diffusion equations [20]. Because equation (3.3) includes the planar propagation speed $V_{0}$, we refer to it here as an MCF-like model or approximation. Equation (3.3) is, therefore, used as a heuristic approximation, valid when the front remains locally close to a planar wave. Near the critical bottleneck width, finite front thickness and boundary-induced distortion may limit its quantitative accuracy.

The MCF approximation is useful to understand the wave blocking seen in our model. Consider a wavefront in figure 4A. Initially, the front is planar so $\kappa (\vec{x},t)=0$. Recall that curvature is the inverse of the radius, and the planar front has an infinite radius, that is, zero curvature. Thus, the normal speed V of the second wave is identical to $v_{2}$. When the wave is about to exit the bottleneck (see figure 4A, t=50), the wavefront will take a round shape whose radius should be around $W_{SB}$. This reflects the fact that the front is perpendicular to the boundary under the zero-flux boundary condition. By assuming an MCF model with the constant curvature $\kappa =1/W_{SB}$, we obtain the condition for the second wave to be blocked as

(3.4) $$\frac{1}{W_{SB}}>v_{2}.$$

The MCF model predicts a negative relationship between $\omega_{c}$ and $v_{2}$ (a curve in figure 2C). This prediction agrees well with our numerical results when $\omega_{c}$ is large but shows noticeable discrepancies when $\omega_{c}$ is small. In cases where the MCF approximation works well ($Z^{*}/\theta =28.5$, $W_{SB}=7.6∼\omega_{c}$) (see figure 2A,C), the stationary distribution of $N_{2}$ appeared to have a thin circular transitional layer (figure 7). By contrast, when the MCF approximation breaks down ($Z^{*}/\theta =26$, $W_{SB}=2∼\omega_{c}$), the transitional layer appeared thick and distorted rather than being a perfectly circular arc (figure 4B). This breakdown occurs because the thickness of the transitional layer (i.e. the width of the wave profile) becomes large relative to $W_{SB}$, making it difficult to approximate the full dynamics by the reduced dynamics of a locally one-dimensional wavefront. It is, however, important to note that the thicknesses of the transitional layers for the two cases are similar. They are different relative to the size of the spatial domain (compare scales of figures 4B and 7). When the spatial domain is large enough (i.e. large $W_{SB}$), we conjecture that the MCF approximation is good. When $Z^{*}/\theta$ is small and hence $v_{2}$ is large, it predicts a small $\omega_{c}$ value, which is not large enough for the MCF approximation. This explains why the approximation is not good for small $Z^{*}/\theta$.

**Figure 7.**
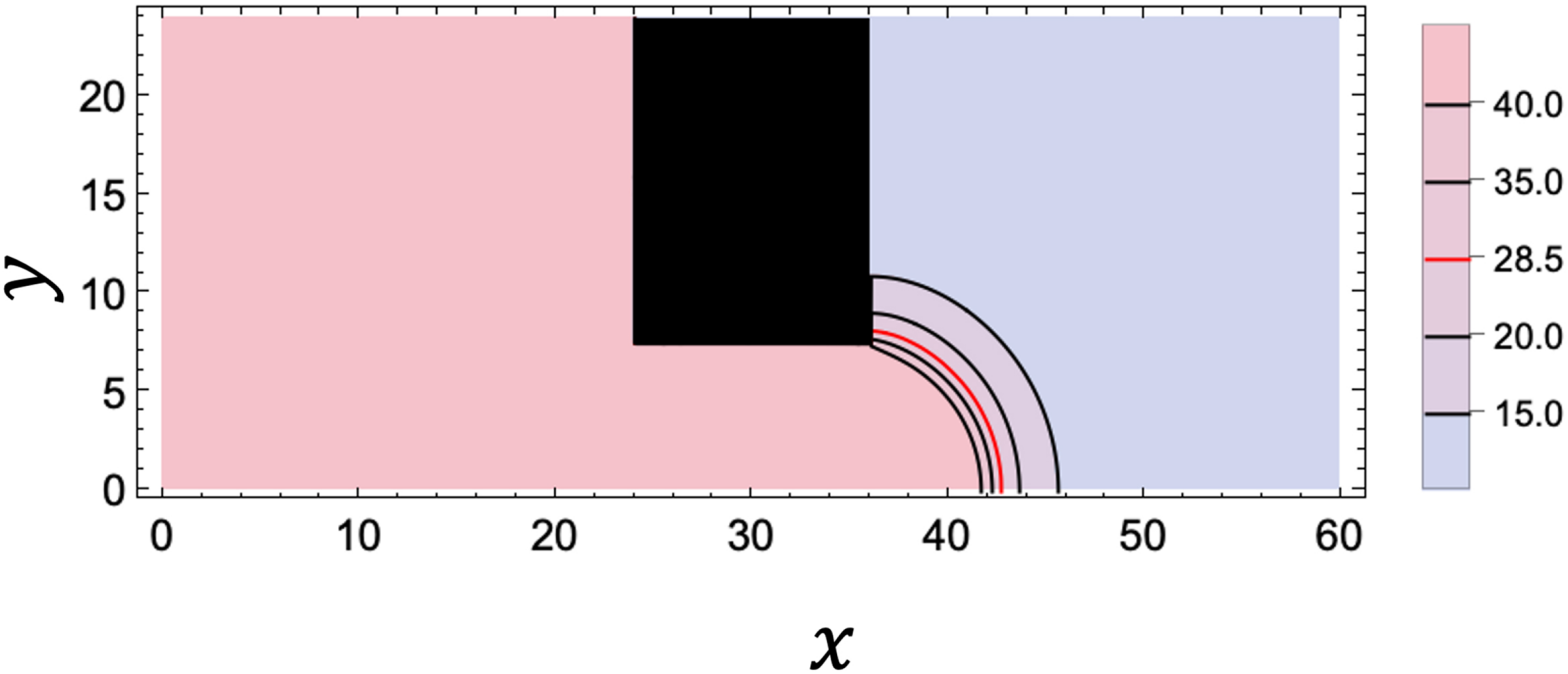
Stable distribution when the MCF model gives a good approximation for $\omega_{c}$. The distribution of $N_{2}$ when $Z^{*}/\theta =28.5$ and $W_{SB}=7.55$ is plotted from the simulation at t=1040, at which the stable condition satisfies (i.e. $\overset{―}{\left|\Delta N_{2}\right|}<10^{-6}$). The domain shape is $W_{1}=24,W_{2}=24,L_{SB}=12,L_{1}=24\;\text{and}\;L_{2}=24$. Parameters are the same as in figure 3 (if not specified).

#### 3.4.2. Robustness of the results

So far we have provided results when parameter values are symmetric between archaic and modern humans. In the supplementary material, we provide additional numerical results showing that blocking occurs when we modify the model to allow asymmetric parameters between the two species. We also argue how the threshold width $\omega_{c}$ should depend on the one-dimensional speed $v_{2}$ in the asymmetric cases.

### 3.5. Speeds and width with units

We have so far shown results after rescaling time and space so that r=D=1. Here, we provide a numerical example when we assume parameter values in kilometres and years. The first wave is of a Fisher–Kolmogorov type, and the (minimum) speed is given by Wakano *et al.* [10] as

(3.5) $$\begin{array}{r} v_{1}=2\sqrt{r(1-b)D}. \end{array}$$

This indicates the speed of geographical spread of modern humans. With our baseline parameter values (b=0.5), we have a unitless speed of $v_{1}=\sqrt{2}$. Assuming that one generation of humans is 15 years and that the population will double every generation under the ideal condition, we have the intrinsic growth rate r=(ln⁡2)/15≈0.0462 $yr^{-1}$. Assuming that the inter-specific competition is half of the intra-specific competition (b=0.5) and that a diffusion coefficient is D=10 $km^{2}$ $yr^{-1}$, we have the speed of the first wave $v_{1}=2\sqrt{r(1-b)D}\approx 0.961$ $km\;yr^{-1}$.

The dynamics of the second wave also depends on the ratio $M_{L}:Z^{*}/\theta :M_{H}$. Using the parameter values shown in table 1, a unitless speed of the second wave $v_{2}\approx 0.3$ is translated into $0.3\sqrt{Dr}\approx 0.204$ $km\;yr^{-1}$ and the critical width $\omega_{c}\approx 2$ is translated to $2\sqrt{D/r}\approx 29.4$ km. When we increase $Z^{*}$ to $14\;km^{-2}$, we have the speed $\approx 0.12\;km\;yr^{-1}$ and the critical width ≈80km. Recall that these values do not depend on the unit of counting population.

**Table 1.** List of parameters and their units. Values are only for the purpose of explanation.

parameter	value	unit
r	0.0462	$yr^{-1}$
θ	0.5	—
b	0.5	—
$Z^{*}$	13	$km^{-2}$
$M_{L}$	20	$km^{-2}$
$M_{H}$	50	$km^{-2}$
D	10	km${}^{2}$ $yr^{-1}$

## 4. Discussion

The prehistoric human spread was shaped by a complex process involving the geographical spread of humans and their culture. In this study, we investigated the impact of spatial bottlenecks on the geographical spread of humans and their culture. A key finding is that spatial bottlenecks do not affect the geographical spread of modern humans (the first wave), but they do hinder the spread of the high-density–high-culture equilibrium and the resulting extinction of archaic humans (the second wave). Spatial bottlenecks slow down or completely block the second wave if they are sufficiently narrow. We characterized the critical width of the bottleneck below which blocking occurs.

### 4.1. Why blocking occurs

The mechanism of blocking of the second wave can be intuitively understood by the effect of the dilution of population density. In our model, a reduction in population size has a negative effect on the number of individuals possessing the skill that enhances carrying capacity. Because the second wave represents a state characterized by high population density and a large number of such skilled individuals, the post-bottleneck dilution of the population is expected to impede a shift to the second wave. This mechanism is incorporated into our model in line with previous studies on the relationship between demography and cultural evolutionary processes, including innovation rates, transmission fidelity and the risk of cultural loss, drawing on ethnographic and archaeological records [6,21–24]. Under the local cultural equilibrium $Z_{i}=\theta N_{i}$, $Z^{*}/\theta$ is the critical population density at which skilled individuals are sufficiently numerous to raise carrying capacity. Archaeologically, it represents a composite threshold for maintaining a demographically beneficial skill repertoire, potentially depending on the multiple cultural factors such as social-learning efficiency, the risk of cultural loss, and technological complexity.

To understand blocking mathematically, let us first consider the second wave with a planar front with a constant speed $v_{2}>0$ (see the red line in figure 4A at t=7). It has a monotonic wave profile as shown in figure 3B. At each location, modern human density is increasing over time. Since the second wave is a bistable wave, the reaction term is negative in front of the wave and positive behind it. The diffusion term can be negative or positive depending on whether the wave profile is convex or concave in the direction of travel. Mathematically speaking, in a planar wave we have $\partial^{2}N_{2}/\partial y^{2}=0$ and thus $\Delta N_{2}=\partial^{2}N_{2}/\partial x^{2}$. The speed $v_{2}$ is determined by the balance between the reaction and the diffusion in the x-direction. This travelling wave has a positive speed because the positive effect from the diffusion (i.e. migration from a high-density area behind) overcomes the negative effect from the reaction (i.e. tendency to fall back to the lower equilibrium density) at all locations in front of the wave.

Now consider a system with a spatial bottleneck. When the wave is about to exit the bottleneck, dilution occurs since the human population also diffuses in the y-direction. This brings an additional effect from the diffusion term that tends to decrease population density. As noted above, reduced population density impedes a shift to the second wave. Mathematically speaking, $\partial^{2}N_{2}/\partial y^{2}$ starts to be negative. This makes the second wave tentatively slow. When all the effects are balanced at all spatial points, there appears a spatially heterogeneous stationary solution and the second wave is blocked (figures 4B and 7).

### 4.2. Implication for mathematics

If the second wave is blocked at the bottleneck while the first wave propagates to the (right) end of the finite spatial domain Ω, it means that the system has a stable heterogeneous stationary solution. Matano [16] mathematically proved the existence of a finite domain Ω in which a stable heterogeneous stationary solution exists in a bistable one-species reaction–diffusion equation with zero-flux boundary conditions. Matano & Mimura [17] obtained the same result for a bistable two-species reaction–diffusion system. For such a solution to exist, Ω needs to be non-convex, which is consistent with our model (figure 1).

In a more recent study, Berestycki *et al.* [25] proved that blocking of bistable waves occurs when they go through an abrupt opening [25]. Furthermore, Etheridge *et al.* [26] studied an Allen–Cahn equation, a reaction–diffusion equation with a bistable reaction term, and showed that the blocking of the bistable wave occurs by the wide opening domain, which is similar to our domain shape. These rigorous results are shown for one-species bistable dynamics. Two-species systems are more complex. For example, even the sign of the velocity of a bistable wave in a two-species Lotka–Volterra reaction–diffusion system is mathematically difficult to determine [27]. Our results provide numerical examples in which blocking occurs in a two-species system.

### 4.3. Boundary conditions and landscape heterogeneity

Geographical barriers such as mountains, deserts, and oceans can be modelled in different ways. In this study, we represented such barriers by zero-flux, or reflective, boundary conditions. Under this assumption, eco-cultural expansion cannot cross the boundary. This formulation may capture situations in which individuals or groups recognize the presence of a barrier and do not attempt to migrate beyond it. However, real geographical barriers are rarely perfectly impermeable. Because zero-flux boundaries exclude any cross-boundary leakage, the barrier effect studied in this model might be stronger than what it was in reality. If individuals instead tend to move along the boundary, this would require a boundary rule combining no normal flux with tangential movement, or an explicit assumption of anisotropic dispersal near the boundary. Examining how partial reflection and directional movement along barriers affect eco-cultural wave propagation is, therefore, an important direction for future work.

We also assumed that habitats inside the boundary are homogeneous and equally habitable. In reality, however, landscapes contain low-habitability regions such as mountains, deserts or other harsh environments. Such landscape heterogeneity in habitability could be modelled through spatial variation in ecological parameters, such as carrying capacity, mortality or dispersal rates. Spatial heterogeneity in habitability can affect the dynamics of eco-cultural range expansion since ecological dynamics is locally variable. Investigating the effect of ecological heterogeneity is another important direction for future work.

### 4.4. Implication for archaeology

The results of this study on eco-cultural spatial dynamics offer new insights into the spatial distribution patterns of archaeological cultures, particularly in regions characterized by geographic bottlenecks. One such region is the area around the Sinai Peninsula, which serves as a land bridge connecting the African and Eurasian continents. This corridor is well known as a major route for repeated human dispersals out of Africa since approximately two million years ago. In the case of Lower Palaeolithic dispersals, the Oldowan and Acheulean lithic assemblages found in the Levant have been interpreted as evidence of lithic technologies that spread from Africa alongside archaic hominin groups [28–30].

Regarding the dispersals of anatomically modern humans (AMHs), the Nubian Levallois technology has been suggested to represent the range expansion of AMHs from Africa to southwest Asia during the middle phase of the Middle Palaeolithic [31–33]. Lithic assemblages characterized by this technology are found across northeastern Africa, the southern Levant and the Arabian Peninsula. In addition, archaic human populations may have coexisted with AMHs in the Levant during this phase [34,35], potentially corresponding to the first wave in our eco-cultural range expansion model.

By contrast, the distribution of Early Upper Palaeolithic bladelet technology shows a sharp boundary between northeastern Africa and the Levant. This technology, known as the Ahmarian industry, developed in the Levant by AMHs and subsequently spread into the Sinai Peninsula [36–38]. However, it did not extend into northeastern Africa, where other lithic technologies existed that resembled earlier ones in the Levant, such as blade technology [39]. This distributional boundary stands in contrast to the widespread presence of similar bladelet technologies across other regions of West Asia, such as the Zagros and the Caucasus, as well as in Europe, where they are represented by the Aurignacian industries [5,40,41].

As suggested in our previous studies, bladelet technology in the Early Upper Palaeolithic can be regarded as a skill that became widespread during a phase of high population density, as predicted by our eco-cultural range expansion model [10]. The halt in its spread at the Sinai Peninsula appears to support the model's prediction that the cultural phase of high population density tends to terminate at the end of a spatial bottleneck.

Another region potentially relevant to our eco-cultural model is the Iberian Peninsula, where the presence of Protoaurignacian or Early Aurignacian bladelet assemblages is sparse and remains debated, particularly in areas south of the Ebro River basin [42]. The Protoaurignacian and Early Aurignacian are lithic industries attributed to AMHs and are characterized by bladelet technology [43]. Sites associated with these industries are abundant in the Franco-Cantabrian region, with their southern distribution bounded by the Cantabro-Pyrenean cordillera [44]. The delayed spread of Aurignacian bladelet technology into the Iberian Peninsula has been linked to the prolonged presence of Neanderthals using Middle Palaeolithic technology, as proposed in the Ebro Frontier model [45]. However, the validity of this model remains contested, particularly regarding the chronological evidence for the appearance of Aurignacian assemblages and the persistence of Middle Palaeolithic sites [42,46,47].

Here, we do not intend to engage in the chronological debates surrounding the Ebro Frontier model, but rather focus on its proposition that the delayed appearance of Aurignacian bladelet technology was due to physical barriers such as the Cantabro-Pyrenean cordillera and the Iberian Systems, which impeded the terrestrial movement of Palaeolithic foragers [42]. This idea is broadly relevant to the findings of our study, as our model suggests that spatial bottlenecks formed by mountainous terrains can constrain the spread of the high-population-density cultural phases, such as those represented by the Protoaurignacian and Early Aurignacian.

### 4.5. Further perspective

More broadly, our model shows how feedback between population density and cultural accumulation can shape spatial propagation. Higher density supports cultural accumulation, while accumulated culture increases carrying capacity; spatial dispersal determines whether this feedback can become established beyond a bottleneck. Related spatial eco-evolutionary models have similarly shown that coupling between population state and spatial dynamics can generate non-trivial outcomes [48].

In this study, we examined a scenario of the Middle-to-Upper Palaeolithic cultural transition. Nevertheless, our eco-cultural range-expansion model can also be applied to other major transitions in which demographic and cultural processes interact. A prominent example is the Neolithic revolution—the transformation from hunting and gathering to farming and herding. Previous models have distinguished between demic diffusion (the spread of farming through migrating populations) and cultural diffusion (the adoption of farming by resident hunter–gatherers), typically formalizing these processes as monostable reaction– diffusion fronts (Fisher-type fronts) [49,50]. However, the Neolithic transition may also exhibit characteristics indicative of bistable dynamics: farming could be sustained only above a critical population or knowledge threshold. Such threshold effects are readily accommodated within our eco-cultural framework. Extending this approach to the Neolithic case may thus provide novel insights, for example into the ways spatial bottlenecks constrained—or facilitated—the spread of early farming societies.

## Supplementary Material



## Data Availability

The code used for this study is available at Zenodo [51]. Supplementary material is available online [52].
