# Supplementary material for: The effect of spatial bottleneck on human eco-cultural range expansion
Source: J R Soc Interface. 2026 Oct 21;23(243):20251007. doi: 10.1098/rsif.2025.1007 (PMC13682887; doi:10.1098/rsif.2025.1007)
Supplement: Supplementary file 1 [file rsif.2025.1007.zip › rsif.2025.1007/rsif20251007_si_001.pdf]

Supplementary Information for "The effect of spatial bottleneck on human eco-cultural range expansion"

Ryuichi Kumata[1,2], Seiji Kadowaki[3], and Joe Yuichiro Wakano[4]

[1]CEFE, CNRS, Montpellier, France
[2]Institute of Tropical Medicine, Nagasaki University, Japan
[3]The University Museum, The University of Tokyo, Japan
[4]School of Interdisciplinary Mathematical Sciences, Meiji University, Tokyo, Japan

In the main text, we assume that both archaic and modern hominoids have the same cognitive capability and ecological characteristics (i.e., the same reproductive rate, mobility, and learning parameters). In general, these characteristics might be different for each species. In this Supplementary Information, we present the asymmetric model and its numerical analysis.

S1. Asymmetric model

The asymmetric model is

$$\frac{\partial N_1}{\partial t} = r_1 N_1 \left(1 - \frac{N_1 + bN_2}{M_1(Z_1)}\right) + D_1 \Delta N_1, \tag{S1}$$

$$\frac{\partial N_2}{\partial t} = r_2 N_2 \left(1 - \frac{N_2 + bN_1}{M_2(Z_2)}\right) + D_2 \Delta N_2, \tag{S2}$$

$$\frac{\partial Z_1}{\partial t} = r_1 Z_1 \left(1 - \frac{N_1 + bN_2}{M_1(Z_1)}\right) - \gamma_1 Z_1 + \delta_1 (N_1 - Z_1) + D_1 \Delta Z_1, \tag{S3}$$

$$\frac{\partial Z_2}{\partial t} = r_2 Z_2 \left(1 - \frac{N_2 + bN_1}{M_2(Z_2)}\right) - \gamma_2 Z_2 + \delta_2 (N_2 - Z_2) + D_2 \Delta Z_2 \tag{S4}$$

where the asymmetric carrying capacities are given by

$$M_i(Z_i) = \begin{cases} M_{H,i} & Z_i > Z_i^* \\ M_{L,i} & Z_i \le Z_i^* \end{cases} \tag{S5}$$

Rescaling of time and space

To simplify the analysis, we introduce the following rescaling:

$$\tilde{t} = r_1 t, \quad \tilde{x} = \sqrt{r_1/D_1}\, x, \quad \tilde{y} = \sqrt{r_1/D_1}\, y \tag{S6}$$

This rescaling normalizes the system so that $r_1 = D_1 = 1$. With this rescaling, we have the scaled equations:

$$\frac{\partial N_1}{\partial \tilde{t}} = N_1 \left(1 - \frac{N_1 + b_{12}N_2}{M_1(Z_1)}\right) + \tilde{\Delta} N_1, \tag{S7}$$

$$\frac{\partial N_2}{\partial \tilde{t}} = \tilde{r}_2 N_2 \left(1 - \frac{N_2 + b_{21}N_1}{M_2(Z_2)}\right) + \tilde{D}_2 \tilde{\Delta} N_2, \tag{S8}$$

$$\frac{\partial Z_1}{\partial \tilde{t}} = Z_1 \left(1 - \frac{N_1 + b_{12}N_2}{M_1(Z_1)}\right) - \tilde{\gamma}_1 Z_1 + \tilde{\delta}_1 (N_1 - Z_1) + \tilde{\Delta} Z_1, \tag{S9}$$

$$\frac{\partial Z_2}{\partial \tilde{t}} = \tilde{r}_2 Z_2 \left(1 - \frac{N_2 + b_{21}N_1}{M_2(Z_2)}\right) - \tilde{\gamma}_2 Z_2 + \tilde{\delta}_2 (N_2 - Z_2) + \tilde{D}_2 \tilde{\Delta} Z_2 \tag{S10}$$

where

$$\tilde{r}_2 = r_2/r_1, \tilde{\gamma}_i = \gamma_i/r_1, \tilde{\delta}_i = \delta_i/r_1, \tilde{D}_2 = D_2/D_1 \tag{S11}$$

To simplify the notation, the tilde symbol is omitted hereafter.

Reduced asymmetric system

If the acquisition and loss of cultural skills are faster than the time scale of human reproduction and death, the skilled densities quickly converge to a local cultural equilibrium

$$\begin{aligned} Z_1(x, y, t) &= \theta_1 N_1(x, y, t) \\ Z_2(x, y, t) &= \theta_2 N_2(x, y, t) \end{aligned} \tag{S12}$$

where

$$\theta_i = \frac{\delta_i}{\delta_i + \gamma_i} \tag{S13}$$

represents the proportion of skilled individual of species i at the cultural equilibrium. Moreover, if eq.(S12) is satisfied at $t = t_1$, then it holds for any $t \geq t_1$ (i.e., it is an invariant manifold of the original four-variable system). To see this, we define

$$w_i(x, y, t) = Z_i(x, y, t) - \theta_i N_i(x, y, t), \tag{S14}$$

and assume that at time $t = t_1$

$$Z_i(x, y, t_1) = \theta_i N_i(x, y, t_1), \tag{S15}$$

holds for any x and y. Substituting eq.(S1-S4) into r.h.s of the following identity

$$\frac{\partial w_i}{\partial t} = \frac{\partial Z_i}{\partial t} - \theta_i \frac{\partial N_i}{\partial t}. \tag{S16}$$

we have

$$\frac{\partial w_i}{\partial t} = r_i w_i \left(1 - \frac{N_i + b_{ij}N_j}{M_i(Z_i)}\right) + D_i \Delta w_i + (\delta_i + \gamma_i) w_i. \tag{S17}$$

Since $w_i = 0$ is a trivial solution, this means that if

$$Z_i = \theta_i N_i \tag{S18}$$

holds initially, then it remains true for all time.

Here, we assume this cultural equilibrium and consider the following two-variable system

$$\frac{\partial N_1}{\partial t} = N_1 \left(1 - \frac{N_1 + b_{12}N_2}{M_1(\theta_1 N_1)}\right) + \Delta N_1, \tag{S19}$$

$$\frac{\partial N_2}{\partial t} = r_2 N_2 \left(1 - \frac{N_2 + b_{21}N_1}{M_2(\theta_2 N_2)}\right) + D_2 \Delta N_2. \tag{S20}$$

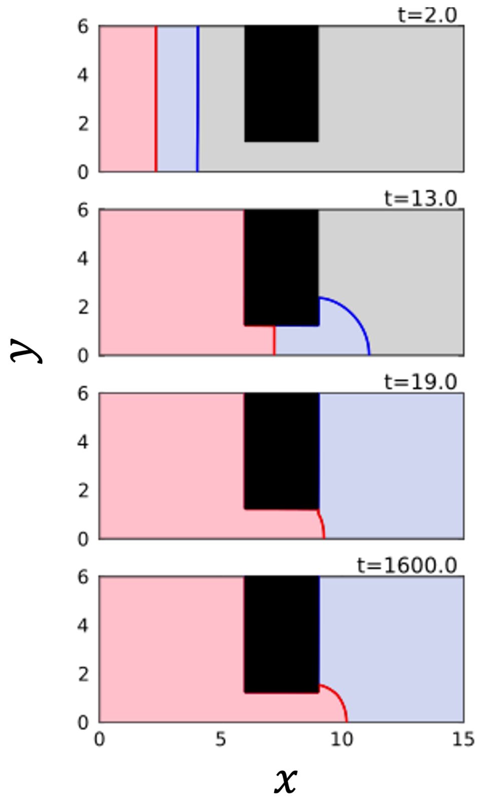

Figure S1: Snapshots of the distributions. Each panel shows the regions where the modern human density is $N_2 > Z^*/\theta$ (light red region), $1 < N_2 < Z^*/\theta$ (light blue region), and $N_2 < 1$ (light gray region) at the indicated time. Parameters: $D_2 = 1.05, Z_2^* = 13, b_{12} = b_{21} = 0.5, \theta_1 = \theta_2 = 1/2, M_{H,1} = 50, M_{L,1} = 20, M_{H,2} = 52.5, M_{L,2} = 21$. The domain shape: $W_1 = 6, L_1 = 90, L_{\rm SB} = L_2 = 0$

S2. Existence of blocking in the asymmetric model

To confirm the robustness of wave blocking, we investigated the eco-cultural range expansion when diffusion coefficients and carrying capacities are different between modern and archaic humans. We consider the case that the moderns have 5% larger carrying capacities and also have 5% larger diffusion coefficient. In the asymmetric case, we found that the second wave can be blocked by a sufficiently narrow bottleneck (Fig. S1).

S3. Threshold values of the asymmetric model

We numerically investigated the dynamics of the asymmetric model by varying all the biological and cultural parameters for the modern humans. In all sets of parameter values studied, we found the threshold width ω_c (Fig. S2). We also measured the one-dimensional speed v_2 of the cultural wave over the same parameter ranges (Fig. S3). To check the relationship between ω_c and v_2, we combined the data calculated above (Fig. S4). Each colored points indicate the data (threshold values and wave speed) from the scan for the indicated parameters. All data but D_2 shapes the single curve similar to $\omega_c = 1/v_2$. This suggests that the threshold values are determined by one-dimensional wave speed and our MCF approximation at least qualitatively captures the relationship. However, only the data from the scan for D_2 deviates from this relationship (black points in Fig. S4), suggesting that the MCF approximation cannot

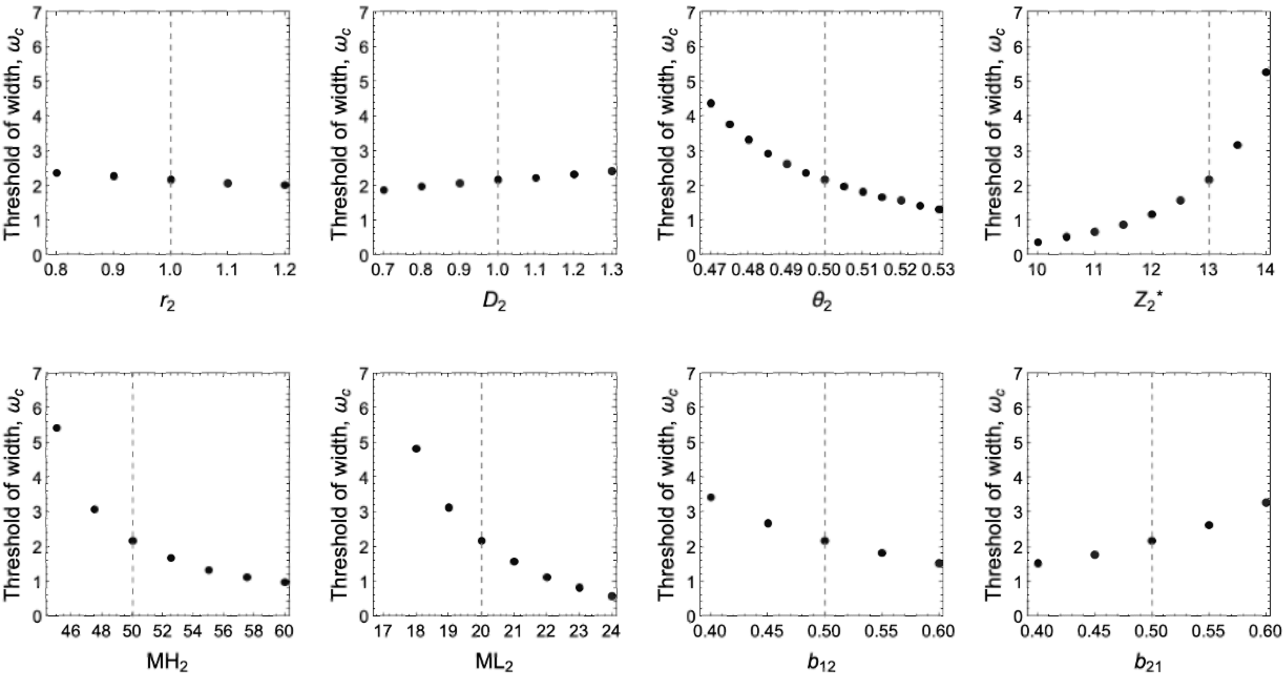

Figure S2: A. the dependency of ω_c on asymmetric parameters. Black dot indicates the data point of ω_c when one parameter of species 2 is varied. Dashed line indicate the symmetric case. The basic domain shape: $W_1 = 24, W_2 = 24, L_{SB} = 12, L_1 = 24, L_2 = 24$. Basic symmetric parameters: $r_2 = 1, D_2 = 1, Z_1^* = Z_2^* = 13, b_{12} = b_{21} = 0.5, \theta_1 = \theta_2 = 1/2, M_{H,1} = M_{H,2} = 50, M_{L,1} = M_{L,2} = 20$.

be applied to the case with heterogeneous diffusion parameters.

The failure of a simple mean-curvature-flow-like approximation under unequal diffusion coefficients can be understood from the linear eikonal equation for reaction–diffusion fronts (Kuramoto, 1980; Löber et al., 2014). When a front is only weakly curved, it can be approximated locally by a one-dimensional traveling wave propagating in the normal direction. Under this approximation, the normal velocity of the front is written as

$$V(\vec{x}, t) = V_0 - \alpha\kappa(\vec{x}, t), \tag{S21}$$

where V_0 is the velocity of the corresponding planar traveling wave and $\kappa(\vec{x}, t)$ is the local curvature of the front. According to Löber et al. (2014), for a multi-species reaction–diffusion system, α is given by

$$\alpha = \frac{\langle W^\dagger, \mathbf{D}\mathbf{U}' \rangle}{\langle W^\dagger, \mathbf{U}' \rangle}. \tag{S22}$$

Here $\mathbf{U}(\xi)$ is the one-dimensional traveling-wave profile, ξ is the coordinate in the propagation direction. $\mathbf{U}'(\xi)$ is the derivative of the profile by ξ and $\mathbf{D}$ is the diffusion matrix whose diagonal elements are the diffusion coefficient of each species. $W^\dagger(\xi)$ is a function calculated from the traveling wave solutions that depends on the parameters. The brackets denote the inner product over the traveling-wave coordinate, including summation over all components:

$$\langle \mathbf{A}, \mathbf{B} \rangle = \int_{-\infty}^{\infty} \mathbf{A}(\xi)^T \mathbf{B}(\xi)\, d\xi. \tag{S23}$$

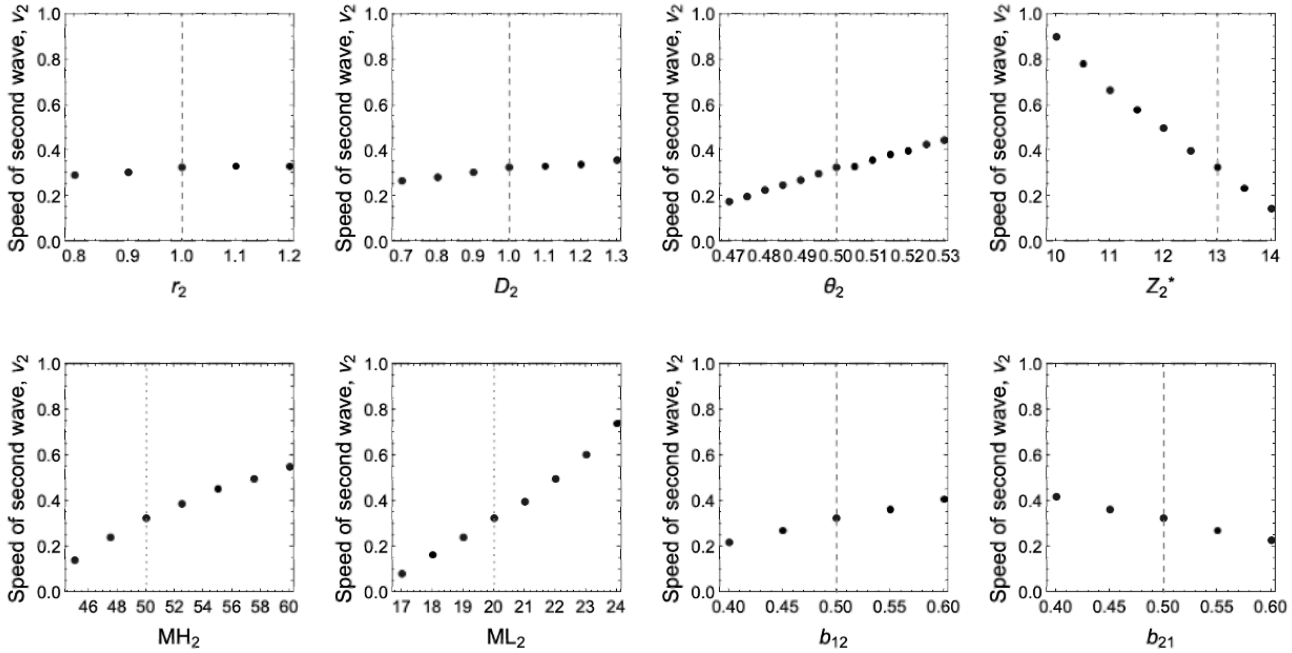

Figure S3: The point is the speed of second wave, c, which is numerically determined from the simulation in one-dimensional space. The points are obtained by the combination of ω_c and c for different z^*/θ. The domain shape and parameters is the same in Fig. S2.

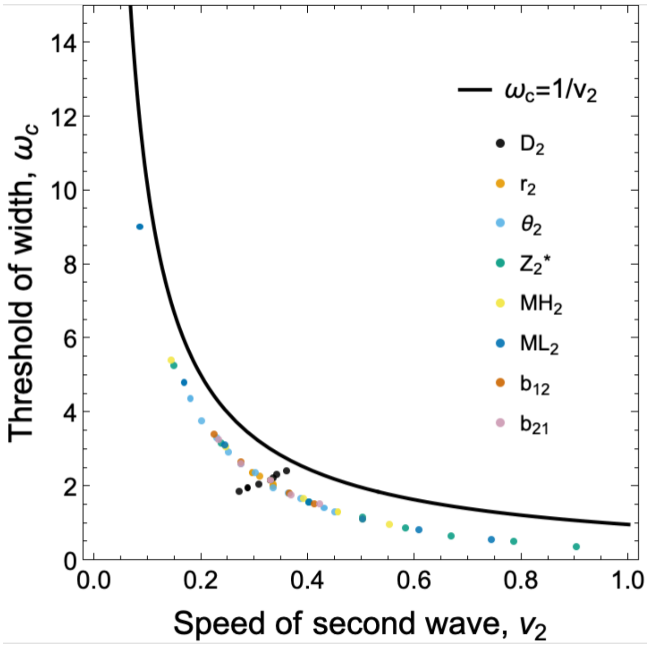

Figure S4: The relationship between ω_c and the speed of wave. Black line is given by the mean curvature flow model, $\omega_c = 1/v_2$. The points are obtained by the combination of ω_c and c from the data in Fig. S2 and Fig. S3. Each color indicate the parameter that is varied.

Symmetric and asymmetric diffusion. In the symmetric diffusion case considered in the main text, all components diffuse with the same coefficient. In particular, when $\mathbf{D} = \mathbf{I}$, the curvature coefficient reduces to

$$\alpha = \frac{\langle W^\dagger, \mathbf{D}\mathbf{U}' \rangle}{\langle W^\dagger, \mathbf{U}' \rangle} = \frac{\langle W^\dagger, \mathbf{U}' \rangle}{\langle W^\dagger, \mathbf{U}' \rangle} = 1. \tag{S24}$$

Thus, the eikonal-curvature relation reduces to

$$V(\vec{x}, t) = V_0 - \kappa(\vec{x}, t). \tag{S25}$$

This explains why, under symmetric diffusion, the relationship between ω_c and v_2 follows the trend predicted by the MCF model presented in main text.

In contrast, when different components diffuse at different rates, α is no longer fixed to unity. Instead, it depends on the diffusion matrix $\mathbf{D}$ and on the internal structure of the traveling wave. Therefore, the blocking condition is expected to be

$$V_0 - \alpha\kappa \simeq 0, \tag{S26}$$

rather than on $V_0 - \kappa \simeq 0$. This provides a possible reason why asymmetric diffusion changes the observed relationship between wave speed and the blocking threshold.

References

Kuramoto, Y., 1980. Instability and turbulence of wavefronts in reaction-diffusion systems. Progress of Theoretical Physics 63 (6), 1885–1903.

Löber, J., Martens, S., Engel, H., 2014. Shaping wave patterns in reaction-diffusion systems. Physical Review E 90 (6), 062911.
